# Legislation for advancing women’s leadership in the health sector in India and Kenya: a ‘law cube’ approach to identify ways to strengthen legal environments for gender equality

**DOI:** 10.1136/bmjgh-2023-014746

**Published:** 2024-07-17

**Authors:** Mireille Evagora-Campbell, Sapna Kedia, Henry Owoko Odero, Radhika Uppal, Sally Atieno Odunga, Tusharika Mattoo, Patricia Blardony Miranda, Sonja Tanaka, Sylvia Kiwuwa-Muyingo, Ravi Verma, Sarah Hawkes, Kent Buse

**Affiliations:** 1 Global Health 50/50, Cambridge, UK; 2 International Center for Research on Women (ICRW), New Delhi, India; 3 African Population and Health Research Center, Nairobi, Kenya

**Keywords:** Accountability, Decision Making, Global Health, Health policies and all other topics, Kenya

## Abstract

**Objectives:**

This paper examines the availability of legal provisions, or the lack thereof, that support women to progress equitably into leadership positions within the health workforce in India and Kenya.

**Methods:**

We adapted the World Bank’s *Women, Business and Law* framework of legal domains relevant to gender equality in the workplace and applied a ‘law cube’ to analyse the comprehensiveness, accountability and equity and human rights considerations of 27 relevant statutes in India and 11 in Kenya that apply to people in formal employment within the health sector. We assessed those laws against 30 research-validated good practice measures across five legal domains: (1) pay; (2) workplace protections; (3) pensions; (4) care, family life and work–life balance; and (5) reproductive rights. In India, the pension domain and related measures were not assessed because the pension laws do not apply to the public and private sector equally.

**Results:**

Several legal domains are addressed inadequately or not at all, including pay in India, reproductive rights in Kenya and the care, family life and the work–life balance domain in both countries. Additionally, we found that among the Kenyan laws reviewed, few specify accountability mechanisms, and equity and human rights measures are mainly absent from the laws assessed in both countries. Our findings highlight inadequacies in the legal environments in India and Kenya may contribute to women’s under-representation in leadership in the health sector. The absence of specified accountability mechanisms may impact the effective implementation of legislation, undermining their potential to promote equal opportunities.

**Conclusions:**

Government action is needed in both countries to ensure that legislation addresses best practice provisions, equity and human rights considerations, and provides for independent review mechanisms to ensure accountability for implementation of existing and future laws. This would contribute to ensuring that legal environments uphold the equality of opportunity necessary for realising gender justice in the workplace for the health workforce.

**Primary source of funding:**

Bill & Melinda Gates Foundation (INV-031372).

**WHAT IS ALREADY KNOWN ON THIS TOPIC**

- There is evidence that legal measures can have a positive impact on women’s economic opportunities and financial security. While equality of opportunity in the workplace is contingent upon a range of factors, the law plays a critical role upon which the systems and structures for equality within (and outside) the workplace rest. The 2023 World Bank’s Women, Business and Law (WBL) review found that, on average, globally, a woman has three-quarters of the legal rights of men in the domains it assesses, that is, mobility, workplace, pay, marriage, parenthood, entrepreneurship, assets and pensions. Such legal discrimination inhibits equal labour force participation of women and deprives women of leadership opportunities at the workplace, including in the health sector.

**WHAT THIS STUDY ADDS**

- Our study adds to existing knowledge in three ways. First, the study builds on the WBL review by reviewing additional good practice legal provisions in the existing domains and by adding a new legal domain that is relevant to women in the world of work, namely reproductive rights. Secondly, it uses a ‘law cube’ approach to delve deeper into the legal domains of workplace, pay, pension, care, family life and work-life balance and reproductive rights. To assess comprehensiveness, we examined the contents of laws within these domains that impact women’s leadership opportunities within the health sector workplace, as well as accountability and equity provisions embedded within these laws. Thirdly, we reviewed laws that were not assessed in the WBL review, introducing additional national-level laws as well as sub-national level laws to our sample. We found that several legal domains are addressed inadequately or not at all, including pay in India, reproductive rights in Kenya and the care, family life and work-life balance domain in both countries. Equity and human rights measures and accountability mechanisms are mainly absent from the laws assessed in both countries. To realise gender justice, ensure equality of opportunity, and promote women’s leadership in the workplace, these legal shortcomings need to be.

**How this study might affect research, practice or policy?**

- The study underscores the critical role of law in promoting gender equality in the workplace, particularly in the health sectors of India and Kenya. It reveals significant gaps in legal protections against gender based discrimination, advocating for comprehensive legal reforms to support women's career progression. For researchers, this study offers a foundational analysis for further exploration of gender equitable legal frameworks. Policymakers can use these insights to design robust laws addressing work-life balance and caregiving responsibilities. Practitioners and leaders are encouraged to align workplace policies with legal mandates, promoting inclusivity and equitable career opportunities for women.

## Introduction

The law underpins and shapes the structures and environments that determine people’s life chances, including their opportunities to enter and advance equitably within the workforce. Legal equality between men and women has been correlated with greater equity in employment opportunities and outcomes and women’s economic empowerment.^1^ The relationship between law and economic equality between men and women is, however, complex. For example, even where provisions to promote and ensure gender equality are comprehensively addressed in the law, implementation depends on a range of factors including social norms and systems of enforcement—meaning the law may not translate from paper to practice.^2^ Further, in settings of legal pluralism, customary or religious laws may over-ride the implementation of more gender-equal formal laws.^1^

Despite these caveats, there is evidence that legal measures can positively impact women’s economic opportunities and financial security. For example, laws that ensure equality in women’s land rights have been instrumental in promoting women’s empowerment.^3^ In the workplace, laws promoting equality and non-discrimination have tended to focus on equal pay, maternity protections and laws prohibiting workplace bullying and sexual harassment. Nonetheless, despite decades of legal reform to promote workplace equality in many countries, women still do not have career equity and are less likely to reach leadership positions than their male colleagues, including in the health workforce.^4–6^

The growth of legal scholarship through a feminist lens highlights the role that women’s unpaid domestic labour plays in driving workplace inequality.^7^ This, in turn, opens a range of additional considerations for how the law could be used to respond to the specific needs of those workers (usually women) who shoulder a double burden, through flexibility in working hours and access to social support for childcare or elder care, for example.^8^

While recognising that equality of opportunity in workplaces is contingent upon a range of factors (as outlined in the accompanying scoping review in this BMJ Collection),^9^ the law plays a fundamental role as the bedrock upon which the systems and structures for equality (inside and outside the workplace) can be built and accountability for equality enhanced.

### Why gender equality in health sector leadership matters

Fairness in opportunities for leadership across genders—fostered by equitable career structures and environments—is a fundamental principle of rights-based gender justice for women in the workforce. Women have rights to equal participation in public life and the spaces of decision-making power. As highlighted in an accompanying paper in this collection,^10^ there are a range of global commitments and accountability mechanisms, including in the Sustainable Development Goals, to promote these rights. Beyond fairness, a more instrumentalist argument seeks to highlight empirical evidence of why gender equality matters. Women’s leadership in the health sector may have a measurable impact on promoting equity in health outcomes.^11^ For example, stronger representation of women in political leadership at the national level has been associated with more equitable public health agendas which focus on women, children and sexual and reproductive health and rights^12^ and at the subnational level on enhanced antenatal care and immunisation and improved neonatal mortality outcomes.^13^

### Gendered inequalities in the health workforce of India and Kenya

Organisations operate with what have classically been called ‘inequality regimes’^14 15^ where societal divisions along gender, class and racial lines are replicated in the workplace. The health workplace is no exception. Women comprise 67% of all health workers and 90% of nurses in the 132 countries reporting sex-disaggregated workforce data.^12^ Women’s participation in spaces of decision-making within global health, however, is much lower.^16^ For example, only 32% of chief delegates to the World Health Assembly in 2023 were women,^17^ and 33% of a sample of 200 global health organisations have not had a woman board chair or chief executive officer in the past 5 years.^18^

The health workforce in India and Kenya follows this global pattern of occupational segregation and under-representation of women in leadership. In India, women make up 29% of medical doctors, 80% of nurses and nearly 100% of unpaid government Accredited Social Health Activists, according to 2017–2018 estimates.^19^ Nursing staff and community health workers are given limited access to decision-making and formal leadership positions throughout the course of their careers.^20^

Gender and professional cadre similarly intersect to influence women’s access to leadership roles in the health sector in Kenya.^21^ In Kenya, medical doctors, who are disproportionately men, are preferentially appointed to leadership positions.^22 23^ Women make up the largest proportion of health sector professionals (58%) in Kenya. Within the health sector, nursing is the only professional health cadre with more women (70%) than men, while other cadres are highly dominated by men.^24^ A study conducted in Kenya’s health training institutions found that men held 62% of the faculty positions.^25^ Thus, despite the Kenyan Constitution explicitly mandating that a single gender should not constitute more than two-thirds of both appointed and elected leadership roles,^26^ particularly within the public sector which comprises a significant portion of the health sector workforce, large gender inequalities continue to persist.^24–26^

### Building on the World Bank’s Women, Business and Law body of work

The annual World Bank Women, Business and the Law (WBL) report details the extent of legal discrimination that inhibits the equal labour force participation of women.^27^ The legal provisions (measures) identified by the WBL report are selected for their association with indicators of women’s economic empowerment, such as employment and business ownership, as well as their recognition in the Convention on the Elimination of All Forms of Discrimination against Women (CEDAW) and International Labour Organization conventions.^27^

The 2023 WBL review finds that, on average, globally, a woman has three-quarters of the legal rights of men in the following domains: mobility, workplace, pay, marriage, parenthood, entrepreneurship, assets and pensions.^27^ Analysis of WBL panel data across 190 countries has found that lower rates of legal discrimination (i.e. a smaller gap in the legal rights available to women compared with men) are associated with ‘fewer women in positions of vulnerable employment and with greater political representation for women’—associations that hold when controlling for overall gross domestic product per capita and for changes within countries as well as comparisons between countries.^1^ The authors of the WBL study conclude that ‘gendered laws matter for economic outcomes’ and ‘measures of legal discrimination are informative about women’s actual status in the economy’.

**Figure 1:**
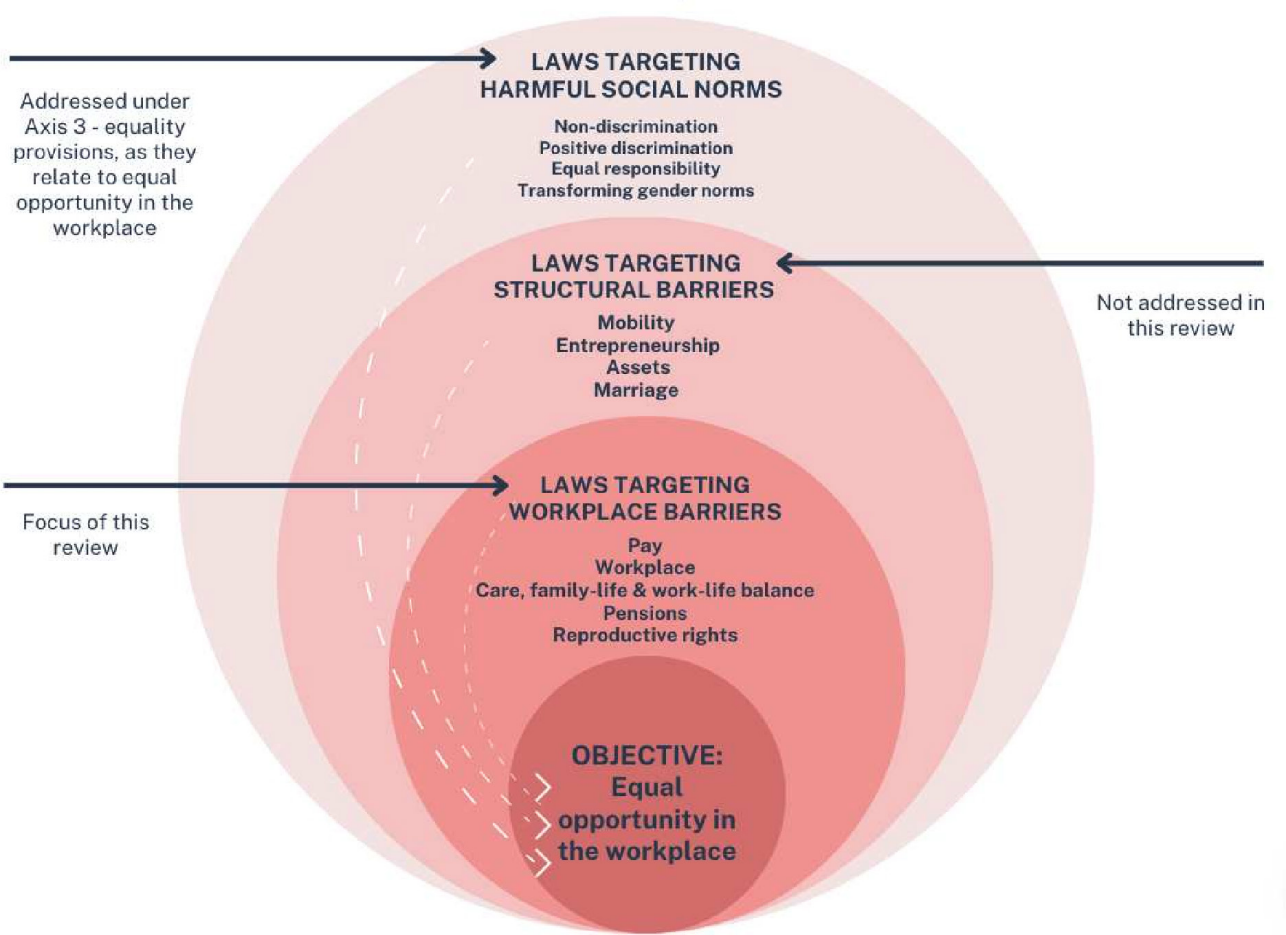
Conceptual framework: legal environments influencing equal opportunity in the workplace.

Our review builds on the WBL analysis, since it has a high degree of legitimacy globally and was relevant to what we wanted to examine. Our focus on laws allowed us to take forward the WBL analysis findings in a more comprehensive way. We do this, by delving deeper into a subsection of these legal domains in India and Kenya, adding additional legal provisions under these domains (see figure 1) and reviewing national laws not included in the WBL’s sample. We also included subnational-level laws given that certain legislative powers are held by state and county governments in India and Kenya, respectively (for at least some of the legal domains under review). We have focused on the following domains present in the WBL work: parenthood, pay, pensions, workplace, meaning we did not look at the domains of assets, entrepreneurship, marriage, mobility. We added a domain on laws relating to sexual and reproductive rights. The rationale for this approach is that we were not seeking only to show how the law impacts women’s employment (WBL focus) but were focused also on the provisions in place to promote women’s equality of opportunity once in the workforce—that is, where and how can the law support women’s aims to advance their careers, including to the very top of their profession. The impact of sexual and reproductive health (SRH) across the life-course and in the workplace is fundamental to our understanding of how goals of gender equality are realised —that is, when women’s SRH rights are not protected and fulfilled, then women are unlikely to achieve gender equality in any space, including the workplace.

### Aims of the study

There are different avenues for reaching gender-equal leadership. In some settings, women’s leadership opportunities are enhanced through strategies such as all-women shortlists for jobs or gender quotas. We have not reviewed the effectiveness of either of these approaches, and instead focus on the question of equality of opportunity along a career pipeline—in recognition that supporting equality along the pipeline will enhance the likelihood of equality at the level of leadership. The aim of our study was to examine the legal environments in India and Kenya that may play a role in determining gender-equitable career progression within the health sector.

## Methods

We reviewed and analysed: (1) the presence of legal provisions in India and Kenya to support women in the health sector workforce to progress equally and advance towards leadership positions; (2) how these laws which contain these provisions perform on accountability and equity and human rights considerations; and (3) the presence of legislative gaps and implications for advocacy efforts and legislative reform for enabling and promoting women’s equal participation and leadership in the health sector workforce in these countries. We focused on India and Kenya to benefit from existing research partnerships in these countries. Both countries also serve as examples of large lower middle-income countries with complex health systems and multiple health sector organisations, thus being potential avenues for advancing women’s leadership. Finally, they are also key partner countries of the funding agency for this research.

The scope of our review was set by three parameters:

First, while the law can be defined as ‘legal instruments such as statutes, treaties and regulations that express public policy, as well as the public institutions (eg, courts, legislatures and agencies) responsible for creating, implementing and interpreting the law’,^28^ for the purposes of this study, we focused solely on statutes, that is, formal written enactments that articulate public policy and provide a structuring effect to codes, regulations and frameworks. In India, we additionally included high-authority rules that prescriptively guide the implementation of statutes in the sample, and which contain specific measures relevant to the legal provisions we examine.

Second, we also reviewed subnational-level laws from several purposively selected jurisdictions. In both India and Kenya, certain legislative functions are constitutionally devolved to the state and county levels, respectively.^26 29^ Several laws in India provide for the power to make rules, or by-laws, for the purpose of implementation of acts. Rules are subordinate to acts but are regularly used to supplement or clarify the objective of the main legislation and guide implementation. In India, subnational rules were reviewed for Uttar Pradesh and Bihar. With over 300 million inhabitants, these are among the most populous states in India and have relatively poor health infrastructure and service delivery performance and have poor health indicators in relation to women, that is, limited access to contraceptives, poor spacing between children, high prevalence of anaemia among women, unsafe abortions and so on.^30 31^ In Kenya, the subnational-legal review focused on only one of the five legal domains, namely care, family life and work–life balance, as the only domain that is devolved to the county level under the Fourth Schedule of the Constitution of Kenya (2010).^26^ Four counties in Kenya were purposively selected, representing both rural and urban environments, and limited to counties with relevant subnational laws. These are Kakamega County, Nairobi County, Embu County and Mombasa County. We also reviewed Turkana County and Wajir County but no relevant laws were found.

Third, while community health volunteers^32^ experience barriers to their career progression, we limit the focus of our review to those who are formally employed in either the public or the private sector (only laws applying across both sectors were reviewed). This is because community health volunteers are often not covered by national labour legislation, and therefore the review’s findings will not apply to this important group of workers—a topic to which we return in the discussion.

### Legal domains

We reviewed laws across five domains. Four of these are included in the WBL annual report and one, relating to SRH in the workplace, is introduced in this review (table 1). We introduced additional legal provisions within the four WBL domains where international treaty law or international multilateral standards identify these as good practices (table 1).

**Table 1:** Research-validated good practice legal provisions

Legal domain	Good practice provisions (WBL=from WBL report)
Workplace	A woman can legally get a job in the same way as a man.^WBL^ Discrimination in employment based on gender or sex is prohibited by law.^WBL^ There is positive discrimination in the law on the basis of gender or sex.^72^ Sexual harassment in employment is addressed in the law.^WBL^ Harassment in employment is addressed in the law.^73^ There are criminal penalties or civil remedies for sexual harassment in employment.^WBL^
Pay	The law mandates equal remuneration for work of equal value.^WBL^ A woman can legally work at night in the same way as a man.^WBL^ A woman can legally work in a job deemed dangerous in the same way as a man (including provision of relevant occupational safety measures).^WBL^ A woman can legally work in an industrial job in the same way as a man.^WBL^ The law mandates equal bonus, provident fund, gratuity, medical insurance, etc. The law does not segregate occupations by gender.^74^
Pension	The age at which men and women can legally retire with full pension benefits is the same.^WBL^ The age at which men and women can legally retire with partial pension benefits is the same.^WBL^ The mandatory retirement age for men and women is the same.^WBL^ Periods of absence due to childcare are legally accounted for in pension benefits.^WBL^
Care, family life and work–life balance	Paid leave of at least 14 weeks is legally mandated to be available to new mothers.^WBL^ The law mandates the government to administer 100% of maternity leave benefits.^WBL^ The law mandates paid leave to be available to fathers.^WBL^ The law mandates paid parental leave to be available.^WBL^ The law requires that employers provide childcare or financial support for childcare or that the government provides childcare services.^74^ The law requires that employers provide (affordable, accessible and quality) elder care or financial support for elder care or that the government provides elder care services.^75^ The law protects the right to work leave (as distinct from maternity, paternity and parental leave) for caring responsibilities.^75^ The law protects the right to flexible working (eg, working from home, condensed hours, flexi-working, annual rather than weekly hours, graduated retirement planning).^76^ Surrogate and adoptive parents have equal leave rights to adoptive parents in the law.^76^ The law protects the right to paid or unpaid nursing breaks.^77^
Reproductive rights (not included in the WBL framework)	Dismissal of pregnant workers is legally prohibited.^WBL^ The law protects the right to take leave for pregnancy-related appointments.^78^ The law protects the right to free and safe abortion.^78^ The law protects the right to leave from work following an abortion or miscarriage.^73^

WBL, Women, Business and Law.

Our initial aim was to examine the pension landscape in India. However, the Pension Fund Regulatory and Development Authority Act of 2013, along with other pension-related legislation, governs pensions solely for government employees and is not obligatory for those in the private sector. We therefore excluded the pension domain from our analysis in India.

The WBL project does not include reproductive rights on the grounds that ‘laws on reproductive rights intrinsically treat men and women differently’ and the WBL index assesses laws that perpetuate discrimination between men and women. However, as noted above, given the impact of SRH on women’s overall health and well-being, including in relation to their working lives, we included these laws in our analysis.^1 18^

### ‘Law cube’ approach to analysis

We extracted and analysed data from the range of laws along the three axes of a ‘law cube’, which draws from the ‘policy cube’^33^ methodology of policy content analysis and assesses laws across three axes. The original policy cube was designed to analyse different types of policy, categorised on their level of authoritativeness. Laws were considered to have the highest level of authority. For the purposes of our paper, we limit the use of the cube to analysing performance of codified laws and do not look at the other types of policy documents. The first axis of the ‘law cube’ assesses the ‘comprehensiveness’ of the legislative environment, that is, the number of good practice legal provisions, set out in table 1, that are addressed in the law in each country. Following the WBL approach, we adopted a binary rather than graded approach to assessing comprehensiveness, in that we measure only whether the provision is present or absent. For example, we look at the availability of paternity leave, rather than examining performance of the law on paternity leave.

The second axis of the ‘law cube’ assesses the extent to which the law articulates ‘accountability’ provisions, or measures to promote effective implementation and monitoring of the implementation. The second axis in the original policy cube is that of political salience and effectiveness and comprises policy authority, accountability and budget commitment. In our adaptation of the cube, we do not look at policy authority since the policies we look at are all laws. We also have not included budgets since laws in India and Kenya do not typically make financial commitment to implement them. Our focus is therefore on accountability on the second axis, that is, the laws’ articulation of systems of accountability. Following Williams and Hunt, who argue that accountability procedures should incorporate human rights values of transparency, accessibility and participation, we adopted their three-pronged model of accountability: monitoring, independent review, and corrective or remedial action.^34^ We have assessed whether each law identifies the actors responsible for ensuring implementation or monitoring implementation. We also assessed whether the law assigns public reporting responsibilities, whether the monitoring mechanism operates independently of the government, and whether provisions in the law exist for remedial actions in cases of non-implementation.

On its third axis, the ‘law cube’ examines whether the law contains equity and human rights provisions to promote equal opportunity and participation in leadership. These could include ‘negative’ and neutral measures, such as those that do not attempt to alter the status quo but cushion its impacts on women (e.g. non-discrimination provisions and measures to support women in undertaking their ‘double-burden’ care responsibilities). These could also include ‘positive’ measures to promote equality through positive discrimination efforts to transform harmful gender norms, such as affirmative action commitments aiming to dismantle barriers to women’s advancement (e.g. reservation in jobs).

In assessing the performance of the ‘accountability’ and ‘equity’ axes, we considered provisions found in any of the five legal domains to count towards a law’s overall scoring on these axes (table 2).

**Table 2:** Law cube analysis methodology

Axis	Description	Scoring
Comprehensiveness	Assesses the performance of legislation against good practice (ie, the adapted version of the Women, Business and Law framework)	Number of good practice provisions (see table 1) present in relevant legislation (total n=30 across 5 legal domains)
Accountability	Assesses measures included in the law to strengthen accountability for proper implementation of the law	Implementation mechanism: 0=no specified agency/body/entity/individual/post/jurisdiction is assigned responsibility for implementation of the law 1=a specified agency/body/entity/individual/post/jurisdiction is assigned responsibility for implementation of the law 2=remedial actions if implementation does not occur are outlined Monitoring mechanism: 0=no specified agency/body/entity/individual/post/jurisdiction is assigned responsibility for monitoring implementation of the law 1=a specified agency/body/entity/individual/post/jurisdiction is assigned responsibility for monitoring implementation 2=a specified agency/body/entity/individual/post/jurisdiction is assigned responsibility for monitoring implementation AND one of the following is true: (1) the body is assigned responsibility for reporting in the public domain or (2) the mechanism is independent from the government
Equity and human rights	Assesses norms, values, ideologies and lenses of analysis relating to equality and human rights evidenced in the law	Number of the following equity and human rights provisions that are present in the law: Commitments to anti-discrimination Positive discrimination measures targeting women or other marginalised/under-represented groups (e.g. quotas/targets for representation of under-represented groups) (and the protected characteristics targeted by these measures) Measures to support women in performing their ‘double burden (in relation to reproductive and productive labour) (e.g. creche facilities) Measures to transform harmful gender norms to promote equal sharing of responsibilities between men and women for reproductive and domestic duties such as unpaid care (e.g. paternity leave, wages for housework)

### Sources of laws

We used the WBL (2022) project dataset^35^ which we augmented by searching national legal databases for additional laws that were relevant to any of the five domains we assessed (see table 1). In India, we used the online legal databases Manupatra,^36^ SCC Online,^37^ Indian kanoon,^38^ CaseMine^39^ and India Code,^40^ *The Gazette of India* 41 and the Ministry of Law and Justice website.^42^ In Kenya, we used the Kenya Law Reports website,^43^ which is a repository containing laws of Kenya.

We identified laws from the above sources that apply at the national level or within selected subnational jurisdictions, relate to any legal provision across any of the five domains of law and are applicable to women in the workforce. The included laws are statutes (or a high-authority rule prescribed within the laws, in India), which have been adopted on any date, currently in force and are published in English.

The reason for including laws irrespective of their adoption date stems from the understanding that laws remain on statute books and may be actively or potentially enforced until explicitly repealed. All amendments, substitutions, omissions or additions to the laws included in the sample were examined. We reviewed national and subnational laws in both countries (see online supplemental annex 1).

10.1136/bmjgh-2023-014746.supp2 Supplementary data

### Data extraction, coding and analysis

We used a standardised data extraction table and a score assigned for each axis. Data extraction and scoring were undertaken independently by two reviewers. In instances of discrepancy between reviewers’ scoring, a third reviewer was engaged to establish a consensus on the scoring.

### Limitations to the methodological approach

In line with the WBL methodology, we adopted a binary (i.e. present or not present), as opposed to graded (i.e. scaled performance), evaluation for each legal provision. As acknowledged in the WBL study, this may mean that certain nuances of the laws are not captured,^44^ and two laws may be ‘scored’ equally when in practice one makes stronger provisions for women. Additionally, our methodology did not cover policies, schemes or clarificatory directives, all of which may reflect implementation.We sought to evaluate the formal legal entitlements to gender equality made by the legislative bodies of India and Kenya to identify gaps within the statutory frameworks that may have consequences for women’s leadership in the health sector. Our analysis, like the WBL framework, does not consider customary law or case law that has not been codified. While legislation sets the framework of the law, and case law interprets and fills gaps in legislation, our intent is to provide a clear, concentrated analysis of statutory frameworks as originally enacted without the variable judicial interpretations introduced by customary/case law. We also only examine the law in so far as it relates to people in formal, paid employment because the informal and voluntary health workforce is ordinarily not covered by labour legislation in either country. Finally, while our methodology allows us to identify legislative ‘gaps’, it does not tell us what their consequences for women’s leadership are in practice.

## Results

We located 27 laws (20 national and seven subnational) in India dated 1923–2013 (three from the WBL dataset and 24 identified from other sources) (see online supplemental annex 1). We found relevant subnational-level laws only in the pay domain for both states (Bihar and Uttar Pradesh). We identified 11 laws (seven national and four subnational) in Kenya dated 2006–2017 (four from the WBL dataset and seven identified from other sources) (see online supplemental annex 1). We identified relevant subnational-level laws for all four counties (Kakamega, Embu, Mombasa and Nairobi).

### Comprehensiveness of Indian and Kenyan legislation

In India, the performance of legislation in terms of the number of good practice (table 1) provisions in place is low, with 12 of 26 possible provisions across the four legal domains in place. Workplace and reproductive rights perform well with four out of six and three out of four provisions in place, respectively. In contrast, legal provisions covering care, family life and work–life balance (three of 10 provisions) and pay (two of six provisions) perform less well (see online supplemental annex 2).

10.1136/bmjgh-2023-014746.supp3 Supplementary data

Laws in Kenya contain 19 of the 30 good practice provisions across the five legal domains (see online supplemental annex 2). The legal domains for which performance is high include pay (six of six provisions), workplace (six of six provisions) and pension (three of four provisions). Fewer good practice legal provisions were addressed in the domains of care, family life and work–life balance (three of 10 provisions) and reproductive rights (one of four provisions) compared with other domains.

### Accountability measures

In India, 19 of the 27 laws contain specific elements related to accountability. Of these 19 laws, 17 assign responsibility for implementation to a named agency, body, entity, individual, post or jurisdiction (none of these laws, however, include remedial actions should implementation not occur). 14 of the 19 laws with accountability provisions contain measures to monitor implementation, for example, through systems of mandatory ‘notification’ of employment information by employers to the government (the Employment Exchanges Act, 1959). However, only two laws with monitoring mechanisms make them independent from the government or commit to reporting on non-compliance in the public domain.

In Kenya, four out of 11 laws contain any accountability measures, all of which assign responsibility to an actor, such as an agency or body, for implementing the law. Accountability mechanisms include, for example, requirements that the relevant Ministers establish councils responsible for implementation of the law (the Labour Institutions Act, 2008) or policy frameworks to guide implementation (the Sexual Offences Act, 2006). No Kenyan laws reviewed, contained measures to assign responsibility for monitoring the implementation of the law.

### Equity and human rights provisions

Three laws in the Indian sample contain equity provisions, all of which are commitments to non-discrimination against women. One law (the Equal Remuneration Act) contains non-discrimination measures targeting groups marginalised based on caste. No laws in the sample contain positive discrimination measures.

Three of 11 Kenyan laws contain equity provisions. Among those, all contain non-discrimination provisions. One law promotes positive discrimination measures (through measures to promote representation of young people and people with disabilities on representative and elective bodies), and one law seeks to address women’s double burden of labour (through guaranteeing fully paid paternity leave for fathers). While three laws mention measures targeting protected characteristics beyond gender, none recognise intersecting forms of discrimination. None contain transformative measures to promote equal sharing of domestic responsibilities between women and men.

**Figure 2:**
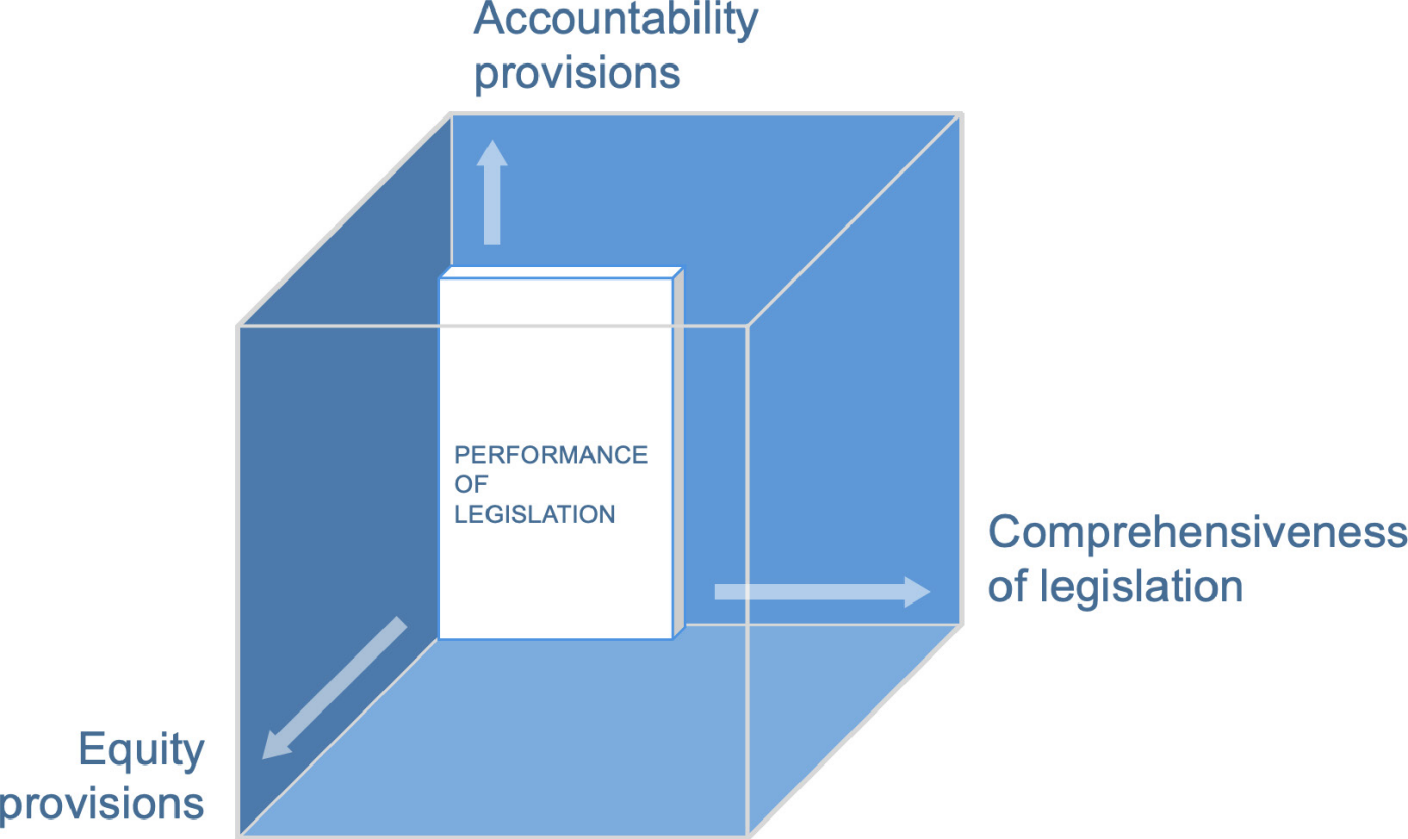
Law cube analysis of Indian legislation : Recommended provisions to promote the advancement of women in the workforce.

**Figure 3:**
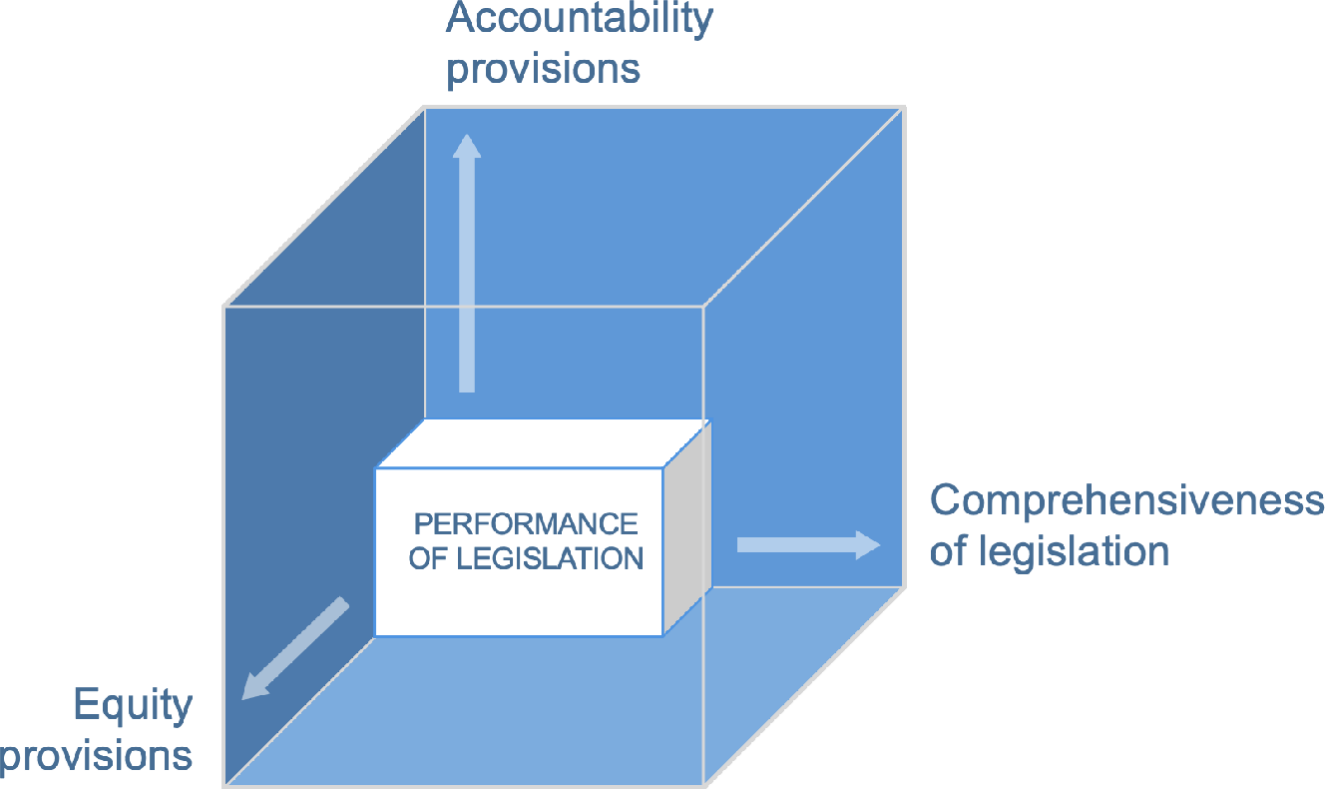
Law cube analysis of Kenyan legislation: Recommended provisions to promote the advancement of women in the workforce.

The findings across all three axes of the law cube are summarised for India in figure 2 and for Kenya in figure 3. The size and proportions of the inner cube represent the performance of legislation along the three axes.

## Discussion

Our review has examined the legal measures that are in place to dismantle barriers to and promote equal opportunities along the career pipeline for women working in formal employment in the health sectors in India and Kenya. Along with examining whether a law is present, using a ‘law cube’ approach permits us to examine the comprehensiveness of the legal environment in each country, as well as accountability and equity considerations associated with each law. Our review has identified areas where more legal and policy attention is needed. Our results show, for example, that laws protecting pay in India, reproductive rights in Kenya, and care, family life and work–life balance in both countries are lacking. Additionally, we find that majority of Kenyan laws reviewed lack accountability mechanisms and that equity and human rights measures are absent from most of the laws assessed in both countries.

The law provides a framework upon which all employees can claim their rights (provided they know these rights and have resources and support to access the courts). This contrasts with, for example, ad hoc organisational workplace policies (e.g. policies to support women experiencing menopause) that may be considered best practice yet be at the discretion of the employer. A methodological strength of our approach is that we build upon a validated framework (WBL). We reviewed additional laws in each of four WBL domains, including subnational and national levels, and added a new domain addressing SRH and rights across the life-course, including in the workplace.^18^

Our review found limited legislative attention to the care, family life and work–life balance domain in both India and Kenya—areas which we have shown to be important determinants of equitable career opportunities (see accompanying paper in this collection—Saville *et al* 9). The focus of legislators has leaned towards expanding the formal (paid) workforce to include women, but without legal interventions to redistribute women’s unpaid domestic labour. The dual burden of production (paid employment) and social reproduction (unpaid domestic labour) experienced by most women hampers their career progression (see accompanying paper^9^), including by contributing to temporary absences, attrition, as well as poorer physical and mental health. This is particularly important in low-income and middle-income countries where women undertake a greater number of unpaid domestic care hours than in high-income countries.^8^ It has been argued^45^ that addressing the legislative gap covering social reproduction requires a ‘reconceptualisation’ of labour law to cover what has previously been termed ‘family law’ and shift the discourse towards ‘the provision of caring rights for those who engage in paid work’. Such an approach would not only raise the status of domestic labour but may also promote the redistribution of domestic care more equally between women and men.^45^ Some measures have been successfully introduced in other jurisdictions. In the European Union, for example, the 2019 Work-Life Balance Directive allows parents and caregivers to request flexible working arrangements to enable them to undertake unpaid care and domestic work as a matter of right.^46^ In the UK, the 2014 Flexible Working Regulations allow employees to apply for flexible working after working for the same employer for at least 26 weeks.^47^ Amendments to the Spanish Civil Code incorporated a commitment into marriage contracts to equitably distribute domestic responsibilities, encompassing the care of children, older individuals and other dependents.^48^ While the effects of these specific laws on women and women leaders have not been studied, existing evidence suggests a positive relation between more equal laws governing the issue of women in the workplace and more equal labour market outcomes relating to gender equality, such as higher participation of females in the labour force and smaller average salary difference between men and women.^1 49^ A study carried out in Taiwan that estimated the effects of legally mandated maternity benefits and working hours restrictions using repeated cross-sectional household survey data found that while working hour restrictions can hinder women’s progress towards equity in the labour market, maternity benefits support women’s efforts to remain in, and advance in, the labour market.^50^ Achieving this level of legal reform will require a concerted effort from a range of stakeholders, including social justice advocacy groups and workers’ rights groups, such as trade unions and professional associations.

The review reveals that important legal entitlements relating to pensions lack universality in India. The pension regulations mandatorily apply only to public sector employees, leaving women in the private sector vulnerable to the discretion of their employers. In contrast, other countries in the region, such as Bangladesh and Pakistan, have pension legislation that covers both formal and informal workers across sectors, though much work remains to be done to ensure that informal workers in these countries are protected by government policy, labour standards and social welfare.^51^ Equal pension rights are especially critical since evidence suggests that women on average earn less than men, have higher caregiving duties, have more career breaks, live longer than men and hence are more likely to live in poverty in old age.^52^

The finding that legal commitments on reporting to independent review bodies are absent from the laws reviewed in Kenya and scarce among Indian laws points to a need for both governments to review legislative processes to ensure that future laws account for robust accountability measures. At the international level, CEDAW provides an example of an independent review mechanism. CEDAW is among the most widely ratified international instruments for women’s rights, seeking to end all forms of discrimination against women.^53^ It addresses women’s rights in the public and private spheres, including the home and the family, and emphasises that state parties are obliged to modify social and cultural patterns to eliminate bias and practices that harm women.^54^ Countries that have ratified the instrument are required to report on their efforts to comply with the obligations contained in the Convention to the CEDAW Committee every 4 years, which can spur country-level legal reforms. However, India and Kenya last submitted reports to the committee in 2012 and 2016, respectively.^55^ Only through regular reporting can governments be held accountable for their international human rights commitments.^56^

Civil society organisations can and do play a pivotal role by submitting shadow reports to monitor government compliance with CEDAW recommendations for their countries. Shadow reports are particularly useful in cases where governments delay self-reporting and can be used to ask follow-up questions to state representatives and apply pressure to improve compliance.^54^ For example, civil society review processes in Latin American countries led to an increase in the media’s mention of CEDAW and greater public interest in compliance with its provisions.^57^ However, these shadow reports are at present produced sporadically, with the last submission for India and Kenya in 2014^58^ and 2017, respectively.^59^ Civil society organisations require resources to support the development of regular independent reports monitoring governments’ compliance with their international legal commitments, including on the legal provisions addressed in this paper.

Despite the critical role played by CEDAW in the struggle for gender equality, its reporting guidelines contain just two-thirds of the 30 legal provisions included in our conceptual framework, with gaps across all five legal domains. For example, the CEDAW guidelines do not require states to report on legislation addressing sexual harassment in the workplace, on rights to work leave for caring responsibilities as distinct from pregnancy or parenting, and on rights to flexible working arrangements.^60^ There is room for stronger advocacy at the global level to ensure that guidelines for country reporting to the CEDAW Committee comprehensively address legal provisions that are critical to equal opportunity in the workplace as the CEDAW process provides a useful, if imperfect, mechanism for accountability for gender equality for women’s leadership—an area which is short of accountability mechanisms generally.^10^

Both the Indian and Kenyan Constitutions contain equality provisions that apply to all national and subnational laws over which they prevail and guarantee fundamental rights,^26 29^ and both countries have ratified the Universal Declaration of Human Rights. In theory, these commitments guarantee rights for all citizens and should promote equity. In practice, however, the incorporation of an equity lens into specific pieces of legislation may also be needed. Our review has found few laws in either India or Kenya contain provisions to promote equity and even fewer do so through commitments to positive action.

A key question for future research is in identifying those factors that have the potential to influence the development and adoption of legal instruments that promote gender equality. For example, what is the evidence for successful legal reform being championed by the action of social and trade union movements pushing for career equality in the workplace.^61^ In India, for example, the women’s movement has been successful in demanding amendments to the county’s divorce, maintenance, domestic violence and maternity laws.^62^ In Kenya, the Federation of Women Lawyers has been successful in advocating for the Adolescent Reproductive Health and Development Policy (2003), the Sexual Offences Act (2006) and the National Reproductive Health Policy (2007).^63^

There is a very large proportion of health workers in India and Kenya who work informally, the majority of whom are women.^64 65^ These informal workers make up the bedrock of the health workforce and yet are often subject to poor and exploitative working conditions for little or even no pay.^64 65^ Critically, these workers are unprotected by labour legislation and the legal provisions we have examined in our study. This leaves them more vulnerable than their formally employed counterparts to inequality and discrimination.

## Conclusion

The law matters for gender equality in the workplace —it provides the bedrock upon which all workers can claim their rights to, among other things, non-discrimination, workplace safety and equality of career opportunity to ensure employees are supported in their career aspirations.

Moreover, the law guarantees fundamental rights and avoids workers being subjected to the vagaries of organisational policy environments provided at the discretion of their employers. This does not obviate the importance of workplace policies, but universality and promotion of equality under the law are vital. In the case of the health sectors in India and Kenya, career progression is generally not equal or equitable. As shown in an accompanying analysis paper by Gideon *et al*,^66^ the occupational gender segregation embedded in health systems produces a workforce where women are responsible for most of the frontline healthcare work, but men occupy the lion’s share of senior leadership. Transforming a workforce depends not only on equitable and fair career structures, but also leaders who use their power to promote collective good and social transformation inside a workplace—an issue explored more fully in an accompanying paper in this collection.^67^

Our study has shown that several legal domains considered critical to promoting equality in career opportunities for women are addressed inadequately or not at all in both India and Kenya, systems of accountability are patchy and considerations of equity are rarely articulated in law. Laws addressing care, family life and work–life balance were lacking in both countries despite being fundamental to women having a fair chance in their careers. Without these laws, women’s double burden of labour (ie, in the paid workforce and in the domestic sphere) will likely continue to be the biggest obstacle to equitable career progression in the healthcare workplace. Addressing these gaps will strengthen the legal foundations for enabling gender equality in the health sector workplace. While this is necessary for career equality, it is likely insufficient.

Progress in the legal domain will also benefit from wider and additional accountability mechanisms—to encourage legislators to act, to encourage the right provisions in legal frameworks and to ensure implementation of the law and legal redress where required. These can be international accountability frameworks as well as organisational-level ones (see companion paper in this collection for an analysis of such frameworks—Buse *et al* 10).

The legal reforms we identified as necessary require legislators to give due recognition to the role of discriminatory gender norms (eg, norms around caretaking and household work) in determining and perpetuating inequities in the health sector workplace.^68^ Accounting for the impact of these norms on women’s work lives will require positive measures, not only anti-discrimination provisions, to be embedded into laws.^69–71^ For example, laws that prohibit gender discrimination in employment will not address the impact of inequitable gender norms without complementary laws making special provisions such as flexible working arrangements to account for gendered caregiving norms. As long as gender norms and their impacts are given only cursory or tokenistic attention in legislative processes, women’s potential to participate and lead in the health sector will remain untapped.

### Dissemination to participants and related patient and public communities

As this is a legal review paper, there are no patients or participants involved. However, we have plans to disseminate our findings to the public via the Global Health 50/50 website and via social media platforms. We plan to conduct in-country dissemination events (roundtables, launch event, media outreach, etc) in India and Kenya to share the findings within the respective countries.

10.1136/bmjgh-2023-014746.supp1 Supplementary data

## Data Availability

All data relevant to the study are included in the article or uploaded as supplemental information.
