# Supplementary material for: Legislation for advancing women’s leadership in the health sector in India and Kenya: a ‘law cube’ approach to identify ways to strengthen legal environments for gender equality
Source: BMJ Glob Health. 2024 Jul 17;9(7):e014746. doi: 10.1136/bmjgh-2023-014746 (PMC11256037; doi:10.1136/bmjgh-2023-014746)
Supplement: Supplementary data [file bmjgh-2023-014746supp002.pdf]

Web Annex 1

Sample of Indian laws containing relevant provisions

**Name of national or sub-national Indian law (n=27)**	**Date adopted**
The Employees Compensation Act 1923	1923
The Payment of Wages Act 1936	1937
Uttar Pradesh Payment of Wages Rules 1936 *(Subnational)*	1936
The Factories Act 1948	1948
The Minimum Wages Act 1948*	1948
The Bihar Factories Rules 1950 *(Subnational)*	1950
Uttar Pradesh Factories Rules 1950 *(Subnational)*	1950
The Employees' Provident Funds and Miscellaneous Provisions Act 1952	1952
The Bihar Shops and Establishments Act 1953 *(Subnational)*	1953
The Employment Exchanges (Compulsory Notification of Vacancies) Act 1959	1959
The Employment Exchanges (Compulsory Notification of Vacancies) Rules	1960
Apprentices Act 1961	1961
The Maternity Benefit Act 1961	1961
Uttar Pradesh Maternity Benefit Rules1961 *(Subnational)*	1961
Uttar Pradesh Dookan Aur Vanijya Adhishthan Adhiniyam 1962 *(Subnational)*	1962
Bihar Maternity Benefit Rules 1964 *(Subnational)*	1964
The Payment of Bonus Act 1965	1965
The Medical Termination of Pregnancy Act 1971	1971
The Payment of Gratuity Act 1972	1972

The Payment of Gratuity Rules 1972	1972
The Payment of Bonus Rules 1975	1975
The Equal Remuneration Act 1976	1976
The Equal Remuneration Rules 1976	1976
Apprentices Rules 1992	1992
The Minimum Rates of Wages in Scheduled Employments 2009	2009
The Sexual Harassment of Women at Workplace (Prevention, Prohibition and Redressal) Act 2013	2013
The Sexual Harassment of Women at Workplace Rules 2013	2013
**Note:** In India, twenty-nine labour laws have been amalgamated in the form of four labour Codes, which are intended to bring in certain reforms. While the Central Government notified the four labour codes in 2019 and 2020, the process of State governments notifying respective rules is still underway and therefore these labour Codes are excluded from our sample.	

Sample of Kenyan laws containing relevant provisions

**Name of national or subnational Kenyan law (n=11)**	**Date adopted**
Sexual Offences Act 2006	2006
Employment act 2007	2007
Labour Relations Act 2007	2007
Occupational Safety and Health Act 2007	2007
Labour Institutions Act 2008	2008
Constitution of Kenya 2010	2010
National Social Security Fund Act 2013	2013
Kakamega County Childhood Development & Education Act 2014 *(Subnational)*	2014
The Embu County Early Childhood Development Education Act 2015 *(Subnational)*	2015
The Mombasa County Childcare Act 2016 *(Subnational)*	2016
The Nairobi City County Children Facility Act 2017 *(Subnational)*	2017
