# Supplementary material for: Legislation for advancing women’s leadership in the health sector in India and Kenya: a ‘law cube’ approach to identify ways to strengthen legal environments for gender equality
Source: BMJ Glob Health. 2024 Jul 17;9(7):e014746. doi: 10.1136/bmjgh-2023-014746 (PMC11256037; doi:10.1136/bmjgh-2023-014746)
Supplement: Supplementary data [file bmjgh-2023-014746supp003.pdf]

Web Annex 2

Performance of sampled laws in India

Legal domain	Legal provisions	Present within legislation (YES/NO)	Relevant law(s)
**Workplace**	Can a woman legally get a job in the same way as a man?	Y	The Employment Exchanges (Compulsory Notification of Vacancies) Act, 1959; The Employment Exchanges (Compulsory Notification of Vacancies) Rules, 1960; Apprentices Act, 196; Apprentices Rules, 1992
	Does the law prohibit discrimination in employment based on gender or sex?	Y	Article 15 of the Constitution
	Is there positive discrimination in the law based on gender or sex?	N	However, certain provisions of the Factories Act, 1948 which are aimed at "protecting women" are provided for at Section 22, Section 27, Section 48, Section 66, Section 87
	Is there legislation addressing sexual harassment in employment?	Y	The Sexual Harassment of Women at Workplace (Prevention, Prohibition and Redressal) Act, 2013; The Sexual Harassment of Women at Workplace Rules, 2013
	Is there legislation addressing harassment in employment?	N	

	Are there criminal penalties or civil remedies for sexual harassment in employment?	Y	The Sexual Harassment of Women at Workplace (Prevention, Prohibition and Redressal) Act, 2013: Indian Penal Code, 1860
**Pay**	Does the law mandate equal remuneration for work of equal value?	Y	The Equal Remuneration Act, 1976; The Equal Remuneration Rules, 1976; The Minimum Wages Act, 1948; The Employees Compensation Act, 1923; The Payment of Wages Act, 1936 U. P. Payment of Wages Rules, 1936; The Minimum Rates of Wages in Scheduled Employments, 2009
	Does the law mandate equal bonus, provident fund, gratuity, medical insurance etc.?	Y	The Payment of Bonus Act, 1965; The Payment of Bonus Rules, 1975; The Payment of Gratuity Act, 1972; The Payment of Gratuity Rules, 1972; The Employees' Provident Funds and Miscellaneous Provisions Act, 1952; The Employees Compensation Act, 1923
	Can a woman legally work at night in the same way as a man?	N	
	Can a woman legally work in a job deemed dangerous in the same way as a man? (this includes provision of relevant	N	

	occupational safety measures)		
	Can a woman legally work in an industrial job in the same way as a man?	N	
	Is there no law that segregates occupations by gender?	Y	The Factories Act, 1948
**Care and work-life balance (adapted from the WBL framework)**	Is paid leave of at least 14 weeks legally mandated to be available to mothers?	Y	The Maternity Benefit Act, 1961
	Does the law mandate the government to administer 100% of maternity leave benefits?	N	
	Does the law mandate paid leave to be	N	

	available to fathers?		
	Does the law mandate paid parental leave to be available?	N	
	Do surrogate and adoptive parents have equal leave rights to biological parents in the law?	N	
	Does the law protect the right to paid or unpaid nursing breaks?	Y	The Maternity Benefit Act, 1961
	Does the law require that employers provide (affordable, accessible, and quality) childcare or financial support for childcare or that the government provides childcare services?	Y	The Maternity Benefit Act, 1961

	Does the law require that employers provide (affordable, accessible, and quality) elder care or financial support for elder care or that the government provides elder care services?	N	
	Does the law protect the right to work leave (as distinct from maternity, paternity, and parental leave) for caring responsibilities?	N	
	Does the law protect the right to flexible working (e.g., working from home, condensed hours, flexi-working, annual rather than weekly hours, graduated retirement planning)?	N	

**Reproductive rights (adapted from the WBL framework)**	Does the law protect the right to take leave for pregnancy related appointments?	N	
	Does the law protect the right to free and safe abortion?	Y	The Medical Termination of Pregnancy Act, 1971
	Does the law protect the right to leave from work following an abortion or miscarriage?	Y	The Maternity Benefit Act, 1961
	Is dismissal of pregnant workers legally prohibited?	Y	The Maternity Benefit Act, 1961, Bihar Maternity Benefit Rules, 1964; UP Maternity Benefit Rules, 1961
Note: As explained in the methods, the pension's domain was not included in the review of Indian laws.			

Performance of sampled laws in Kenya

Legal domain	Legal provisions	Present within legislation (YES/NO)	Relevant law(s)
**Workplace**	Can a woman legally get a job in the same way as a man?	Y	Employment Act, 2007; Constitution of Kenya, 2010
	Does the law prohibit discrimination in employment based on gender or sex?	Y	Employment Act, 2007; Constitution of Kenya, 2010
	Is there positive discrimination in the law based on gender or sex?	Y	Employment Act, 2007
	Is there legislation addressing sexual harassment in employment?	Y	Employment Act, 2007
	Is there legislation addressing harassment in employment?	Y	Employment Act, 2007; Labour Relations Act, 2007; Occupational Safety and Health Act, 2007
	Are there criminal penalties or civil remedies for sexual harassment in employment?	Y	Sexual Offences Act, 2006
**Pay**	Does the law mandate equal remuneration for work of equal value?	Y	Employment Act, 2007
	Does the law mandate equal bonus, provident fund, gratuity, medical insurance etc.?	Y	Employment Act, 2007
	Can a woman legally work at night in the same way as a man?	Y	Employment Act, 2007

	Can a woman legally work in a job deemed dangerous in the same way as a man? (this includes provision of relevant occupational safety measures)	Y	Employment Act, 2007
	Can a woman legally work in an industrial job in the same way as a man?	Y	Employment Act, 2007
	Is there no law that segregates occupations by gender?	Y	
**Pension**	Is the age at which men and women can legally retire with full pension benefits the same?	Y	National Social Security Fund Act, 2013
	Is the age at which men and women can legally retire with partial pension benefits the same?	Y	National Social Security Fund Act, 2013
	Is the mandatory retirement age for men and women the same?	Y	National Social Security Fund Act, 2013
	Are periods of absence due to childcare legally accounted for in pension benefits?	N	
**Care and work-life balance (adapted from the WBL framework)**	Is paid leave of at least 14 weeks legally mandated to be available to mothers?	Y	Employment Act, 2007
	Does the law mandate the government to administer 100% of maternity leave benefits?	N	
	Does the law mandate paid leave to be available to fathers?	Y	Employment Act, 2007

	Does the law mandate paid parental leave to be available?	N	
	Do surrogate and adoptive parents have equal leave rights to biological parents in the law?	N	Employment Act, 2007
	Does the law protect the right to paid or unpaid nursing breaks?	N	
	Does the law require that employers provide (affordable, accessible, and quality) childcare or financial support for childcare or that the government provides childcare services?	Y	The Mombasa County Childcare Act, 2016; The Nairobi City County Children Facility Act, 2017
	Does the law require that employers provide (affordable, accessible, and quality) elder care or financial support for elder care or that the government provides elder care services?	N	
	Does the law protect the right to work leave (as distinct from maternity, paternity, and parental leave) for caring responsibilities?	N	
	Does the law protect the right to flexible working (e.g., working from home, condensed hours, flexi-working, annual rather than weekly hours, graduated retirement planning)?	N	
**Reproductive rights (adapted**	Does the law protect the right to take leave for pregnancy related appointments?	N	

**from the WBL framework)**	Does the law protect the right to free and safe abortion?	N	Constitution of Kenya, 2010
	Does the law protect the right to leave from work following an abortion or miscarriage?	N	
	Is dismissal of pregnant workers legally prohibited?	Y	Section 46 of the Employment Act 2007
