# Supplementary material for: Legislation for advancing women’s leadership in the health sector in India and Kenya: a ‘law cube’ approach to identify ways to strengthen legal environments for gender equality
Source: BMJ Glob Health. 2024 Jul 17;9(7):e014746. doi: 10.1136/bmjgh-2023-014746 (PMC11256037; doi:10.1136/bmjgh-2023-014746)
Supplement: Supplementary data [file bmjgh-2023-014746supp001.pdf]

Reflexivity Statement

This study, titled "Legislation for Advancing Women's Leadership in the Health Sector in India and Kenya: A 'Law Cube' Approach to Identify Ways to Strengthen Legal Environments for Gender Equality," was collaboratively conducted by researchers primarily from the United Kingdom, India, and Kenya, reflecting a diverse international partnership. The research team comprises members from high- (UK) and lower-middle- income (India, Kenya and Phillippines), ensuring a balanced representation of perspectives and expertise. Each author's contribution has been fairly acknowledged, adhering to the guidelines for equitable authorship in global health research.

Reflexive Analysis of Authors' Positions:

1. **United Kingdom:**

Two of the researchers from the UK conceived the study based on previous development of the conceptual framework of a 'policy cube', and brought expertise in policy and legal frameworks, gender studies, advanced research methodologies, and access to research funding. Their perspectives were influenced by working within well-resourced academic and less well-resourced non-governmental environment and having extensive experience in global health and gender equality research. Other UK-based researchers played coordination, research, analysis and writing roles and provided specific inputs on legal measures.

2. **India:**

Indian researchers contributed deep contextual knowledge concerning local legislative processes, cultural nuances, and the healthcare system. Their insights were crucial for tailoring the research to be culturally sensitive and relevant to advancing women's leadership in the health sector. They played a key role in data collection and ensuring the research addressed local gender equality priorities.

3. **Kenya:**

Kenyan researchers provided essential on-the-ground perspectives, access to policy influencers, and understanding of community and legislative dynamics. They played a key role in data collection and ensuring the research addressed local gender equality priorities.

Author Positions on the Research Process:

Design and Methodology: The study design was a collaborative effort, ensuring methodologies were context appropriate and scientifically rigorous. The UK-based researchers contributed to the development of the 'law cube' approach (based on previous work in developing a 'policy cube' for health policy analysis), while Indian and Kenyan researchers ensured these methods were adaptable to local legislative contexts.

Data Collection: Data collection was primarily managed by the researchers in India and Kenya, leveraging their local connections and understanding of legislative and community dynamics. This approach ensured data integrity and relevance. A lawyer in India supported the data collection process.

Analysis and Interpretation: The data analysis was a joint effort, with researchers from each of the countries contributing to the legal analysis. Researchers from India and Kenya provided critical contextual interpretations and worked with UK researchers to build rigour, and

contextualization in the peer-reviewed literature. This collaborative analysis ensured that the findings were both sound and contextually meaningful.

Authorship and Acknowledgments: Joint lead authorship reflects the equal contributions of three researchers from the three participating research teams and acknowledges the importance of equitable partnership. Senior authorship position was determined by the senior author's role in conception, oversight and manuscript writing and revision. Inclusion in authorship was determined based on a mixture of conception, data collection, analysis and writing. All authors have reviewed and approved the final manuscript, ensuring that contributions are fairly recognized. We do not have any acknowledgements in the paper, all contributors are authors.

Ethical Considerations: Ethical approvals were obtained from relevant local and international ethics committees, ensuring compliance with ethical standards. The research prioritised equity by addressing legislative issues pertinent to advancing women's leadership in the health sectors of India and Kenya, ensuring the research outputs are directly beneficial to these communities.
